# Switching off hypertrophy: Conditional siRNA as a precision tool for heart failure therapy

**DOI:** 10.1016/j.omtn.2025.102760

**Published:** 2025-11-10

**Authors:** Takashi Ikenouchi, Eugenio Cingolani

**Affiliations:** 1 Smidt Heart Institute, Cedars-Sinai Medical Center, Los Angeles, CA, USA

## Main text

Since the discovery of small non-coding RNAs as key modulators of gene expression, RNA interference (RNAi) has attracted intense attention and become a widely explored field of research. This ubiquitous gene-silencing mechanism holds tremendous potential not only for fundamental biological investigation but also as a therapeutic modality, as exemplified by the Food and Drug Administration approval of patisiran in 2018 and the ensuing development of multiple RNAi-based therapeutics.^1^ Nevertheless, uncontrolled RNAi activity can produce off-target effects in unintended genes and tissues, making the establishment of precisely regulated systems a long-standing challenge.

To address this issue, several strategies have been developed to externally control RNAi activity, including tetracycline-inducible systems,^2^ protein-dependent switches,^3^ and optogenetic approaches.^4^ More recently, *de novo* designed RNA switches that undergo ligand- or nuclease-induced structural rearrangements have emerged as a rapidly advancing class of tools and already demonstrate clear feasibility.^5^ Of particular importance for safe clinical translation is the ability to condition RNAi on tissue type, cell state, or disease-specific molecular context. Achieving such precise, context-specific control of RNAi is expected to drive major advances in both molecular biology and medicine.

Building on their 2022 work,^6^ in the current issue of the journal, Gokulnath et al. now present a novel riboswitch-based conditional small interfering RNA (Cond-siRNA) platform.^7^ This system incorporates a programmable sensor capable of detecting a predefined biomarker transcript and, in response, activating RNAi against a chosen therapeutic target. As proof of principle, they designed a Cond-siRNA activated by elevated expression of NPPA (encoding atrial natriuretic peptide). Once triggered, the Cond-siRNA silences PPP3CA, which encodes the catalytic subunit α of calcineurin—a mechanistically compelling and therapeutically promising target in cardiac disease, supported by preclinical studies but not yet validated in clinical practice.

The construct is ingeniously designed with three strands: a guide strand and a sensor strand, both hybridized to a central core strand. The 3′ terminus of the sensor strand carries a sequence complementary to the biomarker transcript of interest. Upon binding the biomarker, the sensor strand displaces the core strand, thereby releasing the Guide strand to trigger small interfering RNA (siRNA)-mediated silencing of the preselected target gene. To further enhance functionality, the authors introduced chemical modifications to improve both stability and silencing efficiency, generating first- and second-generation constructs as well as cholesterol-conjugated variants. Importantly, attachment of cholesterol to the sensor strand enabled potent gene silencing without the need for transfection reagents—representing a notable step toward translational applicability.

Biomarker selection was carried out in a phenylephrine (PE)-induced cardiac hypertrophy model. The authors demonstrated that Nppa, a clinically established biomarker of pathological hypertrophy and heart failure, was robustly upregulated following PE treatment, validating its use as a trigger for the sensor system. Leveraging a heart-on-chip platform to recapitulate pressure overload, they further showed that Ppp3ca expression and downstream signaling through the calcineurin-NFAT and mitogen-activated protein kinase/extracellular signal-regulated kinase pathways were markedly suppressed but only under conditions of elevated Nppa. In contrast, conventional siRNA targeting Ppp3ca reduced expression irrespective of Nppa levels. Collectively, these results provide strong evidence that Cond-siRNA enables conditional gene silencing in physiologically relevant contexts.

Calcineurin is a calcium-dependent phosphatase that centrally regulates hypertrophy, remodeling, and cell death and has long been considered a promising therapeutic target in chronic heart failure and cardiomyopathy. Pharmacologic inhibitors such as cyclosporine A and tacrolimus attenuate hypertrophic remodeling by suppressing downstream transcriptional programs, notably NFAT activation in cardiomyocytes.^8^ In the present study, Cond-siRNA treatment reduced cardiomyocyte size and shifted the Myh7/Myh6 ratio, accompanied by upregulation of Murf1, consistent with anti-hypertrophic activity.

However, systemic calcineurin inhibition is associated with significant safety concerns. Broad suppression of calcineurin activity can impair adaptive responses by blocking physiological hypertrophy and can trigger systemic dysfunction due to its widespread expression in the immune system, nervous system, musculoskeletal system, kidney, and pancreas. For instance, the immunosuppressive properties of cyclosporine A are attributable to inhibition of T cell activation. Moreover, pathogenic variants in *PPP3CA* have been linked to neurodevelopmental disorders and epileptic encephalopathy.^9^ These observations underscore the need for selective and context-restricted strategies when targeting calcineurin.

Selective inhibition through a disease- or tissue-specific RNAi system represents an attractive means to mitigate systemic toxicity while improving therapeutic precision. NPPA is predominantly expressed in the heart and is reactivated in the ventricular myocardium under pathological conditions via re-engagement of the fetal gene program, which enhances its value as a cardiac disease-specific trigger. The Cond-siRNA platform is therefore well suited to applications in which a uniquely disease-associated biomarker can be paired with a therapeutic target of interest.

Despite its promise, several steps remain before clinical translation. Constructs composed of complex double-stranded nucleic acid assemblies require rigorous *in vivo* characterization, including assessments of stability, immunogenicity, biodistribution, and metabolic clearance. The reliability of NPPA as a biomarker also warrants careful scrutiny, as its expression can be modulated by comorbidities—underestimated in obesity and metabolic disease or overestimated in renal failure.^10^ In addition, its atrial predominance raises questions about the consequences of atrial PPP3CA suppression. A further concern is dose dependence: in this study, Cond-siRNA at 30 nM appeared to increase Murf1 expression and reduce the Myh7/6 ratio even under conditions where NPPA was likely not elevated. This observation suggests that off-context activity may still occur at higher concentrations, highlighting the importance of titration studies to define an optimal therapeutic window.

Notwithstanding these challenges, the demonstration that a compact engineered small RNA can conditionally activate RNAi and achieve potent, biomarker-dependent knockdown represents a substantial advance in RNAi-based therapeutics. By directly addressing the long-standing problem of off-context activity, the Cond-siRNA platform—if rigorously validated and optimized—has the potential to usher in a new generation of RNA-based therapies, characterized by unprecedented precision and therapeutic impact.

## Declaration of interests

The authors declare no competing interests.
